# Maquet III procedure: what remains after initial complications - long-term results

**DOI:** 10.1186/1749-799X-8-11

**Published:** 2013-05-01

**Authors:** Fernando Fonseca, João Pedro Oliveira, Pinho Marques

**Affiliations:** 1 University Clinic of Orthopedics, Faculty of Medicine, University of Coimbra, Coimbra, Portugal; 2 Orthopedic Department, Centro Hospitalar e Universitário de Coimbra, Praceta Mota Pinto, Coimbra, 3000, Portugal

**Keywords:** Maquet III, Advancing anterior osteotomy of the anterior tibial tuberosity, Patellofemoral osteoarthritis, Long-term treatment

## Abstract

**Background:**

Maquet III procedure, unloved due to its complications (2% to 59%), has been progressively abandoned. At long-term follow-up, what happens to patients with complications that exceeded the initial ones (Acta Orthop Scand 60:20, 1989)? We retrospectively studied patients who were submitted to Maquet III procedure, by functional and radiologic long-term outcomes, in order to determine if this surgery has or has not fulfilled its initially proposed objectives. From 1970 to 1991, 116 patients benefit from the Maquet III procedure. From this, we were able to review in 2011, 23 patients (25 knees) who went through a single Maquet III procedure. Of these patients, 52% were males. Age at surgery was 39.7 ± 11.4, with a postoperative follow-up of 27.2 ± 3.1 years.

**Methods:**

A questionnaire has been prepared for collecting data, and it has been supplemented by clinical records. We evaluated the preoperative complaints, postoperative complications, and range of motion during the recovery time, as well as the postoperative pain-absence period. All patients underwent an objective assessment using the visual analog scale (VAS) at rest and activity, and the Kujala patellofemoral scoring system. A radiological assessment was also made in order to evaluate the arthrosis degree. The bicondylo-patellar angle described by Delgado-Martins (Arch Orthop Traumat Surg 96:303–304, 1980) was used to measure patellar tilt, and the Caton-Deschamps index to calculate the patellar height.

**Results:**

Only one knee had benefited from a total knee arthroplasty (20 years after the Maquet III procedure). Preoperative complains were mainly anterior knee pain, crepitus, and patellar instability. Nowadays, 10 patients (40%) still are pain free. Others had an average period without pain of 19.1 ± 6.1 years. VAS at rest was 1.7 ± 0.7 and in activity 4.4 ± 3.0. KPS was 61.9 ± 22.3 points. X-ray shows that 40% had a Kellgren-Lawrence grade of 1 at the patellofemoral joint.

**Conclusion:**

Maquet proposed this technique for knee-pain relief, maintenance of the knee range of motion, and for slowly progressive osteoarthritic development. Viewed in a dispassionately way, we could notice that the initial objectives of this procedure were completely achieved. A part of 80% of the initial population was lost during follow-up, which may compromise the conclusions, perhaps, it is time to reflect again on this solution, so unloved by so many.

## Background

Isolated patellofemoral osteoarthritis is rare, being present in 3.8% of the osteoarthritic knees [1,2]. Among the causes of chondromalacia and/or patellofemoral osteoarthritis are primary osteoarthritis without trauma history (49%), recurrent patellar instability (33%), chondrocalcinosis (9%), and trauma history (9%) [3].

Ideal treatment for chondromalacia and/or patellofemoral osteoarthritis on younger patients still remains unknown and a controversial topic. Conservative treatment with rest, strengthening of the quadriceps femoralis muscle, and intake of nonsteroidal anti-inflammatory drugs is a successful treatment in a significant number of patients [1,4].

When conservative treatment fails, a surgical approach may be the solution. Several surgical options have been proposed ranging from Pridie's holes [5] to a patellectomy (described by Bentley [4]) as a solution.

Maquet [6] proposed a 2- to 2.5-cm advancing anterior osteotomy of the anterior tibial tuberosity (ATT) for cases of chondromalacia and/or patellofemoral osteoarthritis resistant to conservative treatment. The rationale of this procedure is to augment the angle degree between the patellar and quadriceps tendon, reducing patellofemoral pressure [7]. After an enthusiastic period, many researchers reported a high rate of complications, especially on the healing process, because of the pressure placed under the skin due to the advancement of ATT [8]. In an attempt to reduce this complication, Ferguson et al. [9] suggested a modified procedure, reducing the advance to 1.25 cm.

The aim of this study is to evaluate the long-term results of the patients with osteoarthritis and/or patellofemoral chondromalacia, who underwent a single Maquet III osteotomy [10-13].

## Materials and methods

All surgical records of the Orthopedic Department between 1970 and 1991 were reviewed. Patients (116) who underwent Maquet III procedure for the treatment of chondromalacia and/or patellofemoral osteoarthritis were found. Surgical procedures were performed by or under the supervision of one of the senior authors (PM). Of these patients (*n* = 116), 37 had no record in the National Health Service; 29 cases, in spite of having records, do not had phone numbers for contact to make an interview, and 16 patients had already died. The other patients were contacted by telephone call and were invited for medical evaluation. Six refused because they were suffering from other severe diseases. The remaining patients accepted to come to our hospital, but only 23 were really present for clinical and radiological evaluation. Since two patients were bilaterally operated, we had included 25 knees in this evaluation.

For those who came to the hospital, we prepared a questionnaire to collect data for pain assessment with a visual analog scale (VAS), with the lowest score of 0 that corresponds to ‘pain free’ and highest of 10 which means ‘intolerable pain’. The patients were asked to mark at the line a point that corresponded to the exact level of pain they experienced currently at rest and a point that corresponded to the exact level of pain during daily life activities (DLA). For correlation, we use the Cox assessment scale [14] and the Kujala patellofemoral scoring system [15]. Free-pain period was registered as well as the range of motion.

Additionally, a radiographic study of the knee was made: antero-posterior view with a profile at 30° of flexion and sky view of patella at 30° of flexion. On radiographic evaluation, we tried to identify the existence of patellofemoral conflict, patellar subluxation, and osteoarthritis, classified according Kellgren-Lawrence criteria [16]. Patellar height was calculated using the Insall-Salvati index [17]. Bicondylo-femoral angle, described by Delgado-Martins [18], was utilized to measure the patellar tilt.

We considered an absolute surgery failure when patient is to undergo total knee arthroplasty (TKA) and a relative failure when patient relates pain that affects daily life activities. Patients' records were registered into a database and processed statistically; Chi-squared test was used for the categorical variables, and Student's *t* test for the continuum variables. A *p* value <0.05 was considered statistically significant. All the patients included in the study consented, and an approval from an ethics committee of our Hospital to retrieve the clinical data has been obtained.

## Results

Pain was the most frequent preoperative complaint (*n* = 20; 80%). The main reason for the surgery was patellofemoral osteoarthritis (*n* = 16; 64%), as shown in Table 1.

**Table 1:** Preoperative data

**Demographic data**	**Number**
Age at surgery, mean (years)	39.7 ± 11.4
Age, mean (years)	67.2 ± 11.3
Sex, *n* (%)	
Male	13 (52)
Female	12 (48)
Knee involved, *n* (%)	
Right	16 (64)
Left	9 (36)
Former surgery, *n* (%)	
Yes	5 (20)
No	20 (80)
Main symptom, *n* (%)	
Pain	20 (80)
Crepitus	5 (20)
Diagnosis at surgery, *n* (%)	
Patellofemoral osteoarthritis	16 (64)
Chondromalacia	9 (36)
Re-operation, *n* (%)	
TKA	1 (4)
Sinovectomia	1 (4)
Others	1 (4)

All patients benefitted from an open knee arthrotomy with Maquet III procedure. Five patients benefitted from other complementary surgical procedures (Pridie's holes in two patients, medial meniscectomy in two, and sinovectomy in one). The mean time of hospitalization was 22.8 ± 22.4 days. Only 23.5% of the patients benefitted from physiotherapy procedures.

On the 2011 review, the mean postoperative follow-up was 27.2 ± 3.1 years with a mean pain-free time of 19.1 years. Tables 2 and 3 describe the results. Only one patient benefitted from a TKA 20 years after the primary surgery (4%), and six had pain interfering with DLA (24%). Twelve (48%) of the patients are still pain free since surgery. For those with pain, VAS showed 76% of the patients had no pain at rest (*n* = 19); the remaining 6 patients were distributed between levels 2 and 3. With daily life activity, 76% (*n* = 19) of the patients were also pain-free, 12% (*n* = 3) had pain intensity between 3 and 4, and 12% (*n* = 3) with more than 5. Cox score showed 68% (*n* = 17) as good/excellent, and 32% (*n* = 8) as fair/poor. Kujala patellofemoral scoring revealed an average of 61.8 ± 22.3 points.

**Table 2:** Clinical data

**Postoperative data**	**Number**
Follow-up (mean in years)	27.2 ± 3.1
Time without anterior-knee pain (mean in years)	19.1 ± 6.1
Pain with DLA	
Without pain	12
Light	7
Moderate with physical impact	3
Sports practice	3
VAS at rest (cm)	
0	19
2	3
3	3
VAS with ADL (cm)	
0	19
3	2
4	1
7	3
COX score	
Excellent	9
Good	8
Fair	7
Poor	1
Kujala patellofemoral scoring (mean)	61.8 ±22.3

**Table 3:** Radiologic findings

	**Number**
Patello-femoral osteoarthritis (Kellgren and Lawrence)	
Absent	3
Grade I	10
Grade II	2
Grade III	5
Grade IV	5
Patellar height (Insall-Salvati)	
<0.8	8
0.8 to 1.2	17
Patellar tilt (Delgado-Martins angle)	
<6°	14
6° to 17°	10
>17°	1

**Figure 1:**
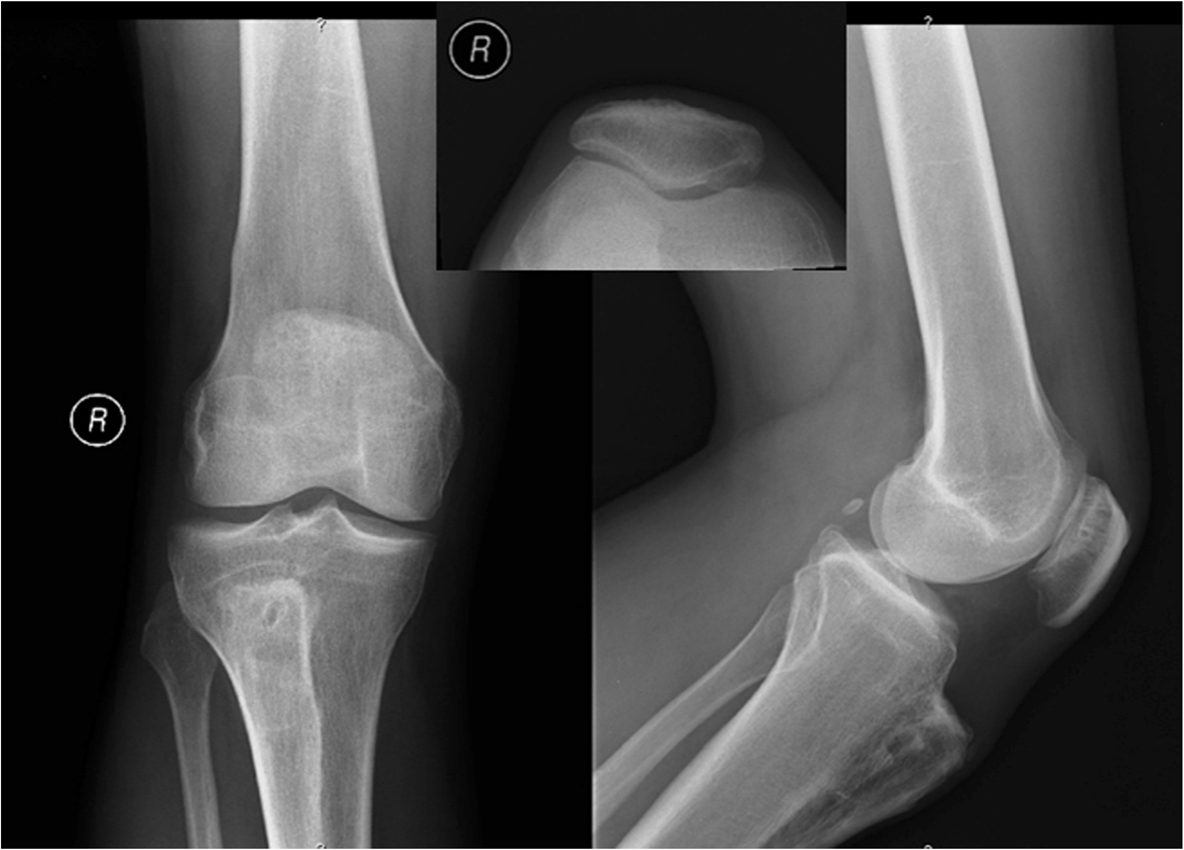
Male, 61 years; excellent Cox score and KPS of 98.

Based on the radiographic study, 48% (*n* = 12) of the patients had an osteoarthritis grade of I-II, and 40% (*n* = 10) had a grade of III-IV. The remaining three (12%) patients still do not have patello-femoral osteoarthritis, independently of the long term follow-up. Patellar tilt measurement using the Delgado-Martins angle [18] shows that 40% (*n* = 10) had a normal angle (6° to 17° at 30° knee flexion), 4% (*n* = 1) had more than 17°, and the remaining 56% (*n* = 14) had less than 6°. Figure 1 shows one excellent case.

The correlation between patello-femoral osteoarthritis and Kujala patellofemoral scoring shows a clinical degradation of quality of life as patients have a grade III or IV radiographic osteoarthritis, as shown in Table 4.

**Table 4:** Correlation of radiological findings versus KPS

**Patello-femoral osteoarthritis**	**Absent**	**Grade I/II**	**Grade III/IV**
Mean Kujala patellofemoral scoring	81.0	64.7	51.7

## Discussion

Patellar chondromalacia was introduced in 1928 by Aleman [19] who described the degeneration of articular cartilage of the patella. Maquet described an original surgical procedure to treat these cases [6]. The treatment [20,21] of chondromalacia and/or patellofemoral arthritis depends on the underlying cause and must be addressed based on that cause. Often, it involves non-surgical measures such as nonsteroidal anti-inflammatory drugs, strengthening of quadriceps femoralis, and lengthening of hamstrings. Surgery is indicated for chronic pain when conservative treatment has failed. The procedures done were cartilage shaving with or without Pridie's drilling [5], Ficat procedure [13], lateral patellar facetectomy [13], patellar realignment, patellofemoral arthroplasty, and total knee replacement [1].

Maquet [6,11] proposed the advancement of the anterior insertion of the patellar tendon at tibial tuberosity, reducing the pressure on the articular patellofemoral joint. Subsequent studies by Kaufer [22], Bandi and Brennwald [10], and Ferguson et al. [9] confirmed Maquet's data. In 1979, Maquet reported a reduction of 50% of the pressure on the patellofemoral joint with a 2 cm advancement of TTA [12]. This advancement was initially associated with a high rate of complications, especially on the healing process [8], as confirmed by Maquet's report (12% of healing complications). Ferguson et al. [9] and Fulkerson and Shea [23] modified the original technique, limiting the elevation to 1.25 cm, reporting only one case of healing problem on 184 patients. Mainly because of healing problems, this procedure has been progressively abandoned. Recent review articles documented a myriad of surgical approaches for this pathology [13], but these procedures do not have a long time follow-up.

What happens to patients with a Maquet III procedure 20 years or more after the surgery? As we know, there is no paper describing such long follow-up result. Maquet [11] reported 95% good to excellent results (*n* = 41 patients), Ferguson et al. [9] 92% (*n* = 48 patients), and Radin [24] 79% (*n* = 42 patients).

Our study has more than 20 years of follow-up (*n* = 25), and even with this long term follow-up, we were able to review by clinical and radiological exams 23 of the 116 cases (19.8%) registered in our hospital. Our long-term results show only 1 (4%) case of absolute surgery failure with 48% (*n* = 12) of excellent results (pain-free with DLA until now) with good Kujala patellofemoral scoring score. Apart from this, the remaining patients who complained of pain tolerate it and had good Cox score.

Have patellofemoral arthroplasty and other procedures resulted to such a performance at long term? Dejour and Allain [3] reported also good results but emphasized that the procedure does not stop the progression of osteoarthritis. We agree with this, but reviewing other procedures such as tibial tubercle medialization, the same authors report a long term development of osteoarthritis. Apart from skin and esthetic problems, patients had good outcomes 20 years or more after a Maquet procedure; this result proves that this procedure is better than other more popular ones.

Maquet III had three purposes: pain relief, maintenance of mobility, and prevention of osteoarthritis. The patients evaluated with a minimum of 20 years, after having pain relief for at least 15 years after surgery, also had good range of motion. Prevention of osteoarthritis was the only purpose that has not been totally achieved, but a delay of 15 years should be taken into account. Taking into consideration the loss of 80.2% of the patients during follow-up may compromise the final conclusions.

First of all, apart from skin complications, evidence supports that Maquet III is a reliable procedure with good results, better than other procedures which have results in less follow-up time. Second, in Maquet III procedure is important to prevent skin complications, as reported on a significant number of cases. Bandi and Brennwald [10] and Ferguson et al. [9] showed that 1 to 1.25 cm is enough to achieve the biomechanics effect proposed by Maquet. But with the advances and the current state of art at plastic surgery, it may be possible to prevent skin complications, avoiding high rates of complications.

## Conclusion

Taking into account the loss of 80.2% of the initial population, in this long-term follow-up, we showed that patients who do not have skin problems after a single Maquet III procedure also have a long free-pain period with functional good outcomes. Avoiding skin problems, Maquet III procedure may be rehabilitated and rise again as a surgical option to treat isolated patello-femoral osteoarthritis.

## Competing interests

The authors declare that they have no competing interests.

## Authors’ contributions

FF designed the study, reviewed the patients, wrote the paper, and supervised the research. JPO reviewed the patients and recorded the patients' data. PM wrote the paper and gave research advices. All authors read and approved the final manuscript.
